# Retinal optical coherence tomography manifestations of intraocular lymphoma

**DOI:** 10.1007/s12348-012-0072-z

**Published:** 2012-04-04

**Authors:** Tin Yan Alvin Liu, Mohamed Ibrahim, Millena Bittencourt, Yasir J. Sepah, Diana V. Do, Quan Dong Nguyen

**Affiliations:** 1 Retinal Imaging Research and Reading Center, Wilmer Eye Institute, Johns Hopkins University, 600 North Wolfe Street, Maumenee 745, Baltimore, MD 21287 USA; 2 Columbia University College of Physicians and Surgeons, New York, NY USA

**Keywords:** Spectral Domain Optical Coherence Tomography, SD-OCT, Intraocular lymphoma, Retinal lymphoma

## Abstract

**Purpose:**

Primary central nervous system lymphoma (PCNSL) is a rare disease. The index report describes a patient with intraocular lymphoma secondary to recurrent PCNSL and corresponding retinal findings on spectral domain optical coherence tomography (SD-OCT).

**Methods:**

Case report.

**Results:**

OCT changes were documented and correlated with the clinical course of intraocular lymphoma progression in the index patient. The OCT changes, manifested as hyperreflective material accumulation in the intraretinal and subretinal pigment epithelial spaces, were caused by lymphomatous infiltration.

**Conclusion:**

SD-OCT can be useful in diagnosing and monitoring the progression or regression of intraocular lymphoma with retinal involvement.

## Introduction

Primary central nervous system lymphoma (PCNSL) is a rare disease, representing 4 % to 6 % of all primary brain tumors and 1 % to 2 % of all extranodal lymphomas [1]. Intraocular lymphoma is a subset of PCNSL that usually involves the retina, the vitreous, or the optic nerve. It is a high-grade, malignant tumor, usually of large B cell histology with the following features: high nuclear to cytoplasm ratios, coarse chromatin, irregular nuclear contours, prominent nucleoli or chromocenters, and occasional presence of mitotic figures [2]. It has been reported that up to 25 % of patients with PCNSL will have vitreoretinal and choroidal involvement [1]. We describe a case of a patient with intraocular lymphoma secondary to recurrent PCNSL with corresponding findings on spectral domain optical coherence tomography (Spectralis HRA+OCT®, Heidelberg, Vista, CA). Following initially successful chemotherapy, relapse was presented as rapid, dramatic visual loss. While fundus examination, fundus photography, and fluorescein angiography showed minimal changes or abnormalities, spectral domain optical coherence tomography (SD-OCT) showed substantial diffuse changes in the fovea, which represented intraretinal and subretinal pigment epithelium (RPE) lymphomatous infiltration.

## Case report

A 72-year-old man presented to the Wilmer Eye Institute in November 2010, complaining of blurry vision in the left eye of 6-week duration. He was diagnosed with PCNSL in 2008, underwent systemic methotrexate and rituximab in 2009, and by early 2010, his disease was in remission. At presentation, the visual acuity with correction in his right eye was 20/25; examination in the right eye was unremarkable. The visual acuity with correction in his left eye was 20/100. Slit-lamp examination in the left eye was remarkable for mutton fat keratic precipitates, 1+ cells and flare in the anterior chamber, and sheets of cells in the vitreous. B-scan ultrasonography of the left eye revealed vitreous opacity; the optic nerve appeared normal. A diagnostic vitrectomy of the left eye confirmed the diagnosis of diffuse, large B cell, intraocular lymphoma, which was cytologically consistent with his previous PCNSL.

Intravenous methotrexate and rituximab were initiated. The patient also received one intravitreal injection of methotrexate in his left eye. During his subsequent follow-up visits in December 2010, February 2011, and April 2011, the patient’s visual acuity with correction in the left eye improved to 20/20 and the vitreous in the left eye remained clear. However, intravenous methotrexate was discontinued in April due to renal function abnormality, and the patient was maintained on an every 2-week rituximab regimen.

**Fig. 1:**
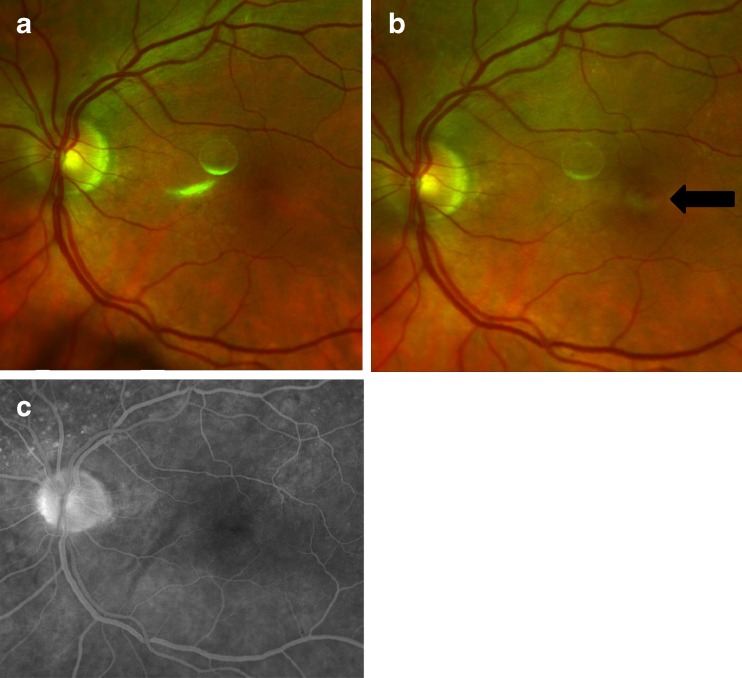
Fundus photography and fluorescein angiography (FA) of the left eye. **a** Color fundus photograph taken in April 2011; visual acuity (VA) was 20/20 and OCT was normal. **b** Color fundus photograph taken in May 2011; VA was 20/250 with abnormal findings in OCT images. The photograph showed a new hypopigmented lesion in the fovea (*arrow*) that was not seen the month prior. **c** Late frame of the FA done in May 2011 showed no leakage in the fovea. The white circular lesions seen between the optic nerve head and fovea in **a** and **b** are imaging artifacts

The patient presented again in May 2011 and complained of decreased vision in the left eye of 2-week duration. The visual acuity with correction in the right eye was 20/20; examination of the right eye was unremarkable. The visual acuity with correction in the left eye was 20/250, decreased from 20/20 one month earlier. The left eye examination was remarkable for presence of keratic precipitates, 1+ cells and flare in the anterior chamber, 0.5+ vitreous haze but no vitreous cells. On fundus photography, there was a new area of hypopigmentation (Fig. 1b) that was not present 1 month earlier (Fig. 1a). On angiography, there was no leakage in the fovea in the late frames (Fig. 1c).

**Fig. 2:**
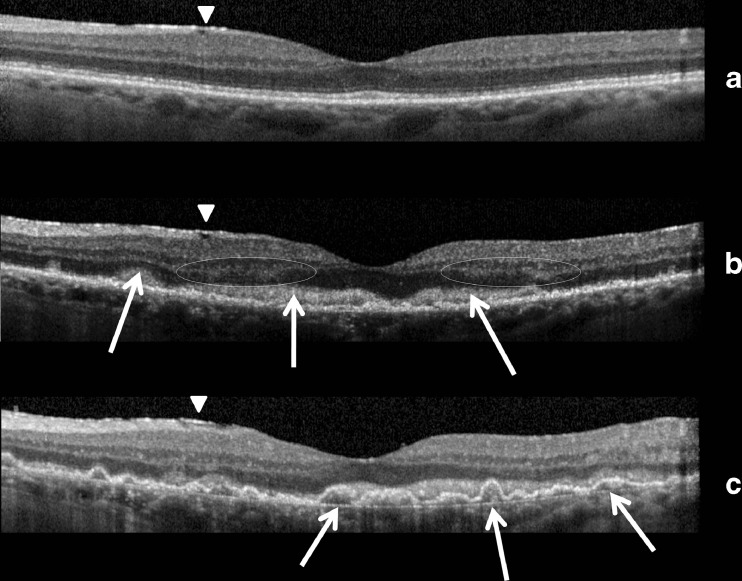
OCT of the left eye. **a** April 2011: normal appearing retinal layers accompanying a visual acuity (VA) of 20/20. There was a layer of hyperreflective material superficial to the nerve fiber layer and nasal to the fovea. It most likely represented epiretinal membrane and remained unchanged throughout the visits (*arrowheads*). **b** May 2011: The patient’s VA decreased to 20/250 with an OCT showing the presence of a moderately hyperreflective material within the outer retina (*arrows*). Of note, there was increased irregularity in the RPE; the IS/OS junction and the ELM could no longer be visualized. The outer nuclear layer and outer plexiform layer appeared to be involved as well (*encircled*). **c** July 2011: In addition to the intraretinal hyperreflective material noted 2 months earlier, the OCT showed newly developed pigment epithelial elevations accompanied by heterogeneous, hyperreflective sub-RPE deposits (*arrows*). We used Spectralis HRA+OCT™ with the Automated TruTrak™ functionality, which allowed automated real-time, point-to-point registration of images taken at different time points. Therefore, the three scans presented in this figure were referenced and represented the same section of the retina

OCT of the left eye fovea revealed the presence of a moderately hyperreflective intraretinal material that was absent 1 month earlier (Fig. 2a). The intraretinal material appeared to be masking the photoreceptor, inner segment/outer segment (IS/OS), and the external limiting membrane (ELM) layers (arrows). The outer nuclear layer and outer plexiform layer were involved as well (circled), but the inner retinal layers were spared (Fig. 2b).

The frequency of prednisolone acetate was increased to six times a day to control the iridocyclitis in the left eye. Over the course of the next 2 months, signs of inflammation in the left eye subsided, and the visual acuity with correction in the left eye improved to 20/40. At that time, OCT from the left eye demonstrated, in addition to the previously noted intraretinal hyperreflective material, newly developed pigment epithelial elevations accompanied by diffuse hyperreflective sub-RPE deposits and an intact Bruch’s membrane (Fig. 2c).

## Discussion

To our knowledge, the index case report is the first to document longitudinal OCT changes, which are temporally correlated with a decrease of visual acuity, in a patient with a history of intraocular lymphoma and retinal involvements. Despite the lack of histopathological evidence from the retina, we believe, given the clinical findings, that the hyperreflective materials shown on OCT most probably represent lymphomatous infiltrations for several reasons.

First, prior case reports have shown that infiltration of lymphoma cells into the retina could cause a whitening of the retina [3]. Such reports could explain the appearance of the new hypopigmented lesion in the left eye fovea (Fig. 1b), which coincided with the decrease in our patient’s visual acuity and appearance of new OCT findings.

Second, Chan and colleagues used a murine model to show that lymphoma cells in the vitreous could migrate through the retina and gather between the neural retina and the RPE [4]. Such collection of cells could explain the presence of the hyperreflective material that masked the ELM and IS/OS junction in the OCT of our index patient (Fig. 2b). We believe that the accumulation of such hyperreflective material, which most likely represented lymphomatous infiltration, resulted in disruption of the IS/OS junction and contributed to the visual decline in our patient.

Third, several histopathological studies of autopsied eyes from patients with primary intraocular lymphoma have revealed the presence of malignant cells between the RPE and Bruch’s membrane [5–7]. The malignant cells are most likely represented as the hyperreflective sub-RPE deposits seen in our OCT, which may have contributed to the newly detected pigment epithelial elevations (Fig. 2c). Furthermore, the migration of malignant cells from the vitreous to the retina, as demonstrated by Chan et al., may explain the appearance of the hyperreflective material in the outer neuroretinal layers before accumulation in the sub-RPE space. However, it does not explain the apparent sparing of the inner retinal layers.

Although other authors have reported OCT findings that they attributed to lymphomatous infiltration [8–12], our index case is the first to document changes in SD-OCT that have correlated with a clinical course of intraocular lymphoma progression. Since we were able to compare subsequent OCT findings with the baseline OCT scan, we believe that the intraretinal and sub-RPE hyperreflective materials were indeed lymphomatous infiltration.

## Conclusion

Intraocular lymphoma may cause visual loss through diffuse retinal infiltration. Spectral domain OCT may provide the necessary resolution to visualize such lymphomatous infiltration, and it can be useful in diagnosing and monitoring the progression or regression of intraocular lymphoma with retinal involvement.
